# Recent Advances in *In-Vitro* Assays for Type 2 Diabetes Mellitus: An Overview

**DOI:** 10.1900/RDS.2020.16.13

**Published:** 2021-05-01

**Authors:** Nazmina Vhora, Ujjal Naskar, Aishwarya Hiray, Abhijeet S. Kate, Alok Jain

**Affiliations:** 1 Department of Biotechnology, National Institute of Pharmaceutical Education and Research-Ahmedabad, India.; 2 These authors contributed equally.; 3 Department of Natural Products, National Institute of Pharmaceutical Education and Research-Ahmedabad, India.; 4 Department of Bioengineering, Birla Institute of Technology Mesra, India.

## Abstract

**BACKGROUND:**

A higher rate of attenuation of molecules in drug discovery has enabled pharmaceutical companies to enhance the efficiency of their hit identification and lead optimization. Selection and development of appropriate *invitro* and *in-vivo* strategies may improve this process as primary and secondary screening utilize both strategies. *In-vivo* approaches are too relentless and expensive for assessing hits. Therefore, it has become indispensable to develop and implement suitable *in-vitro* screening methods to execute the required activities and meet the respective targets. However, the selection of an appropriate *in-vitro* assay for specific evaluation of cellular activity is no trivial task. It requires thorough investigation of the various parameters involved.

**AIM:**

In this review, we aim to discuss *in-vitro* assays for type 2 diabetes (T2D), which have been utilized extensively by researchers over the last five years, including target-based, non-target based, low-throughput, and high-throughput screening assays.

**METHODS:**

The literature search was conducted using databases including Scifinder, PubMed, ScienceDirect, and Google Scholar to find the significant published articles.

**DISCUSSION and CONCLUSION:**

The accuracy and relevance of *in-vitro* assays have a significant impact on the drug discovery process for T2D, especially in assessing the antidiabetic activity of compounds and identifying the site of effect in high-throughput screening. The report reviews the advantages, limitations, quality parameters, and applications of the probed *invitro* assays, and compares them with one another to enable the selection of the optimal method for any purpose. The information on these assays will accelerate numerous procedures in the drug development process with consistent quality and accuracy.

## 1 Introduction

Diabetes is a complex incurable metabolic disorder which is one of the major factors in the rapid surge in healthcare costs [1]. Destruction or dysfunction of insulin-producing pancreatic beta-cells is a vital characteristic of diabetes [2]. Insulin-dependent type 1 diabetes mellitus (T1D) is an autoimmune disorder caused by the demolition of insulin-producing pancreatic beta-cells. Non-insulin-dependent type 2 diabetes mellitus (T2D) is a heterogeneous disorder caused by the dysfunction of insulin-producing beta-cells, resulting in impaired insulin secretion or increased insulin resistance in response to glucose in skeletal muscle, hepatic system, and adipose tissue [3-6]. With an expansion in the pervasiveness of T2D every year, the International Diabetes Federation (IDF) Diabetes Atlas reports that around 463 million individuals were affected by diabetes in 2019, in the age group of 20-80 years. This is expected to increase to 700 million by the year 2045.

**Figure 1.**
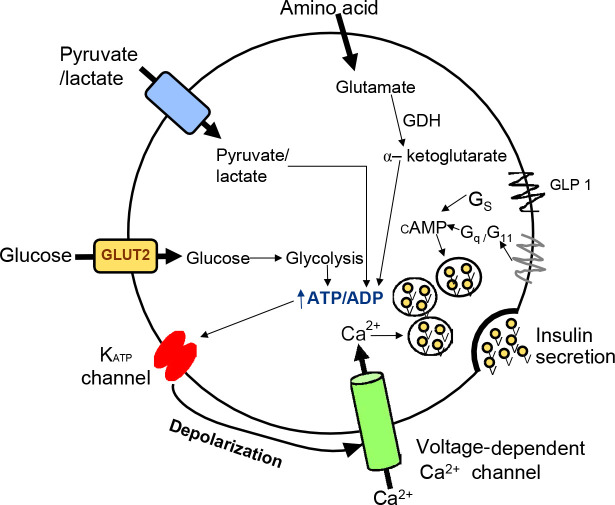
**Schematic representation of the pathway for glucose and hormone in the pancreatic β-cell**. This figure outlines the impact of glucose which increases ATP levels through metabolism in mitochondria. Apart from glucose, pyruvate or lactate and amino acids increase the ATP level which prompts depolarization of **β**-cells via activation of the K-ATP channel-dependent pathway and increases Ca^2+^ levels employing voltage-dependent Ca^2+^ channel which stimulates insulin secretion. Additional pathways that animate insulin secretion via an increase in the levels of cyclic adenosine monophosphate (cAMP) and diacylglycerol (DAG) are also shown in the figure.

As diabetes is a metabolic disease causing serious long-term consequences, it is essential to understand the process of insulin secretion to find an effective treatment. Insulin secretion consists of a multi-stage process in which pancreatic islet cells containing the majority of the β-cells initiate cascading events of insulin secretion in response to elevated blood glucose levels (Figure 1). Insulin-filled secretory granules fuse with the plasma membrane and subsequently secrete the stored insulin. In T2D, insulin secretion and insulin sensitivity may be impaired, eventually leading to hyperglycemia [7].

Several antidiabetic drugs have been introduced to improve insulin secretion and sensitivity and to mitigate the symptoms. However, extensive application of these drugs may cause severe side effects. For instance, metformin, an oral diabetes drug operating at the molecular level, inhibits the mitochondrial respiratory chain in the liver leading to the activation of adenosine monophosphate-activated protein kinase (AMPK), and eventually enhancing insulin sensitivity [7], which causes significant side effects including anorexia, nausea, abdominal discomfort, and diarrhea [8]. Another class of drugs, the sulfonylureas, which is designed to inhibit K-ATP channel activity by increasing the level of cytoplasmic calcium and promoting insulin release, causes significant side effects such as loss of efficacy, hypoglycemia, and weight gain [9].

Therefore, a huge research effort is devoted to the development of new antidiabetic drugs that spare these adverse side effects [7]. The advent of *in-vitro* and *in-vivo* assays for early drug discovery overcomes the challenging task of providing a better molecule which is afflicted with less adverse effects than existing drugs. These studies have become essential to improve knowledge and understanding of pathology and pathogenesis, and to find new therapies against T2D. Animal-based screening of the antidiabetic compounds is expensive and raises ethical issues [10]. Such a model is also not extrapolative to the human response, as animal studies are often poor predictors of human reactions to exposure to drugs. The use of a cell-based *in-vitro* system in scientific research overcomes the constraints of direct *in-vivo* models by being cost-effective and ethical [3, 4]. The *in-vitro* approach is useful as primary screening, which would decrease the considerable load of animal-based studies [11].

During the past few decades, efforts have been made to set up an *in-vitro* insulin secretion assay as an effective research platform. However, only a few of these attempts are at the functional stage today. The underlying problem is the need for standardization of assays because of the inadequate response to glucose by pancreatic beta-cells in T2D and the marked shift in the concentration-dependence curve towards higher sensitivity [5]. To overcome these impediments, research efforts are currently aimed at culturing cell lines correctly so as to provide valuable information concerning the physiological procedure. One of the significant obstacles in conducting this substantial research is the lack of a proper insulin stimulation and insulin secretion assay, which is discussed below in section 3.3 on insulin secretion assays. Many of the available assays are incapable of verifying the outcomes reported in the studies. The reason for this incapability is that critical parameters associated with this assay are avoided, which is also discussed below in section 3.3 on the insulin secretion assay.

These constraints and the escalating cost of managing diabetes and its complications indicate an urgent need to establish an *in-vitro* antidiabetic assay. Reliable methods to estimate insulin secretion need to be established in order to complement knowledge on insulin sensitivity in the understanding of T2D. Currently, the most sophisticated techniques used to measure secreted insulin are radioimmunoassay (RIA) and enzyme-linked immunosorbent assay (ELISA) [8], which provide good reproducibility and higher sensitivity. An additional method currently in use is the measurement of glucose uptake that reflects insulin sensitivity, which may be preferred in the future as it is a nonradioactive method [10]. It is undoubtedly necessary to make pinpoint measurements and to detect small changes in the rate of secretion by incorporating specific steps in *in-vitro* techniques. The following sections explore the key *invitro* screening methods developed as modern drug discovery techniques and their advancement.

## 2 Methods

The scientific articles were retrieved in bibliographic databases, including Scifinder (scifinder.cas.org), PubMed (www.ncbi.nlm.nih.gov/pubmed), and Science Direct (www.sciencedirect.com) with an array of keywords, including “antidiabetic assay”, “phenotypic assay”, “target-based antidiabetic assay”, “insulin secretion assay”, and “cAMP assay”. Articles with the best accordance with the keywords were selected by filtering literature published in the last five years (2015-2019). Only those articles were selected that included assays explicitly used for *in-vitro* techniques and T2D.

**Figure 2.**
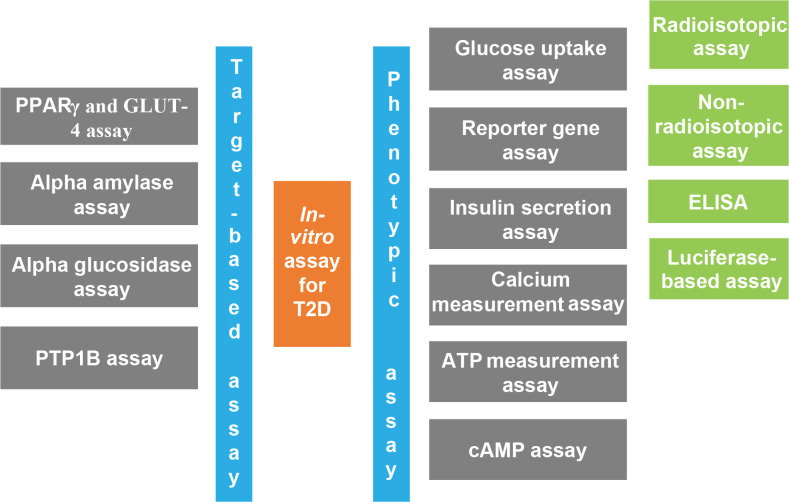
**Overview of the classification of in-vitro assays designed for type 2 diabetes (T2D)**. Phenotypic assays are generally high-throughput cellular assays that measure the levels of various proteins or identify molecules with the ability to alter the phenotype of the cell, whereas target-based assays are designed to target specifically the molecules that are known.

As shown in Figure 2, *in-vitro* assays are classified into two broad categories according to the specificity of the target compound.

## 3 Phenotypic assays

This approach does not rely on a specific drug target or its mechanism or role in disease development. It is the first type of assay that should be performed to check the preliminary antidiabetic activity of any compound, molecule, or extract. Phenotypic types of assays are useful for identifying molecules with the ability to alter the cell phenotype [12, 13].

### 3.1 Glucose uptake assay

Glucose is the principal energy source for different types of cells, and regulation of its uptake by different tissues is critical for glucose homeostasis. Diabetes complications occur as a result of higher blood glucose levels. It is thus necessary to look for antidiabetic effects that cause an increase in glucose uptake such that blood glucose levels are lowered. This assay is primarily useful for the evaluation of antidiabetic activity of compounds or crude extracts through the mechanism of increasing glucose uptake. Glucose transporter 4 (GLUT4) is a glucose transporter, present in adipose tissue, muscle cells, and other tissue; it is responsible for insulin-dependent glucose uptake in these cells. There are various types of glucose uptake assays available that use glucose analog, either radiolabeled or non-radiolabeled [14, 15]. These assays are discussed below.

In the past few years, researchers used the radioisotopic assay as it does not require a highly delicate detector for its detection compared to other assays. This assay includes radiolabeled glucose analogs that mimic the action of glucose and provide a measurable amount of glucose taken up by the cells. These analogs are of two types: phosphorylated and non-phosphorylated, which includes 2-deoxy-D-glucose (2DG) and 3-methylglucose (3MG), respectively. The glucose uptake assay with 2DG is based on the fact that glucose and 2DG are processed similarly in the cell as their phosphorylation is brought about by hexokinase enzyme [16]. The 2-deoxy glucose 6-phosphate (DG6P) is converted from 2DG concentrates in the cell because it cannot convert to an analog of fructose 6-phosphate. 3MG is useful for the evaluation of the initial rate of glucose transport without any interference by the subsequent steps of glucose metabolism. However, due to its speedy equilibration over the cell membrane, it requires a short incubation time. The 2DG assay is preferable over the 3MG because 2DG is phosphorylated into DG6P, a stable analog that accumulates in the cell. Equilibration and radiolabeled isotope efflux will be faster in 3MG than in 2DG. Advantages of assays with radiolabeled analogs include a satisfactory signal-to-noise ratio and a better selectivity than enzymatic or fluorescent-labeled analog. Significant limitations are the cost and handling of radiolabeled compounds [17].

Non-radiolabeled analogues are 6-[N-(7-nitrobemz-2-oxa-1,3-diazol-4-yl) amino]-2-deoxyglucose (6-NBDG) and 2-[N-(7-nitrobemz-2-oxa-1,3- diazol-4-yl) amino]-2-deoxyglucose (2-NBDG) [18]. This assay primarily relies on glucose-6-phosphate dehydrogenase (G6PDH) that catalyzes the formation of 2-deoxy-6-phosphogluconate from 2-deoxy glucose 6-phosphate (DG6P) with the simultaneous formation of the reduced form of nicotinamide adenine dinucleotide phosphate (NADPH) from NADP^+^. Resorufin is formed from resazurin by diaphorase simultaneously with the formation of NADP^+^ from NADPH. The resultant resorufin is a fluorophore measured at the end of the reaction, which is theoretically equivalent to the extent of DG6P. This process is appropriate for high-throughput screening and for the estimation of compounds that may be involved in the regulation of glucose uptake [19]. This assay's major advantages include a lesser time consumption than the radioisotopic assay, and the fact that it is low-cost, reproducible, and nonradioactive. The use of these analogs in the past year has reduced because of difficulties in their fluorescent measurement. However, with photomultiplier technology development, researchers are currently using them more frequently again. The assay can be used in a microplate format for high-throughput screening, and direct imaging of cells can also be performed by using fluorescence microscopy [20]. Limitations involve lower signal-to-noise ratio and lower sensitivity than radioisotopic assays [21-26].

### 3.2 Reporter gene assay

Diabetes and its associated complications are caused by the interaction between genetic and non-genetic factors. At different stages of diabetes, many genes are upregulated or downregulated [27]. Candidate genes include *peroxisome proliferator-activated receptor gamma* (*PPAR*γ), *potassium inwardly rectifying channel subfamily J member 11* (*KCNJ11*), and *ATP-binding cassette transporter subfamily C member 8* (*ABCC8*). The *PPAR*γ gene encodes the target PPARα nuclear receptor for thiazolidinedione. The latter two genes are involved in the regulation of ATP-sensitive potassium channels and are the targets of antihyperglycemic sulfonylureas. Besides these genes, the occurrence of T2D involves many other genes. The expression patterns and changes of these genes are studied today by advanced high-throughput *in-vitro* techniques. There are many types of detection methods of this kind that can be used to examine the effects of compounds on gene expression; one of the evaluation methods is reporter gene detection.

Reporter genes are used to report the desired gene expression [28]. As mentioned above, the genes used to study their appearance can be selected based on their mechanisms and targets. The two types of reporter genes mainly used are countable (by scoring procedures) and selectable. The expression of a countable reporter gene results in a quantifiable phenotype, and it is sensitive and easy to detect. The selectable reporter gene can be used as a qualitative indicator [29]. Primary reporter genes include luciferase, β-galactosidase, green fluorescent protein (GFP), yellow fluorescent protein (YFP), antibiotic resistance genes, and others. Among these genes, luciferase is the most widely used reporter gene because of its highly specific activity and low background noise. With the rapid turnover of the enzyme luciferase and the large, linear dynamic range, these genes are highly reliable for reporter gene assays. The assay begins with the preparation and transfection of the gene construct to obtain stable or transient expression of the gene in the pancreatic β-cell line. The colocalization of the reporter gene and target gene should be confirmed by PCR or DNA sequencing. After this confirmation, the cells should be treated with various concentrations of the test compound in different time periods. Appropriate blanks (untreated), positive controls (cells treated with standard drugs), and negative controls (empty vectors) should be used [30]. After the end of the treatment period, cells should be lysed using an appropriate lysis buffer. To obtain results, cell lysates should be treated with a luciferin substrate. The expressed luciferase causes the conversion and luminescence of luciferin to oxyluciferin. This luminescence can be measured by a luminometer [31]. Gene up- and downregulation can be measured by comparing the luminescence of test and control. Luciferase activity should be normalized by a negative control, which is a transfected blank plasmid [32].

### 3.3 Insulin secretion assay

The insulin secretion assay is a non-target-based assay as it does not cause progress in the direction of a specific target. The definitive objective of the antidiabetic agents is primarily to increase insulin secretion [22]. The chief factor responsible for T2D is impaired insulin secretion. There are different bioassays accessible to assess the secretion level of insulin. Two types are involved predominantly, namely ELISA-based and luciferase-based assay [23].

ELISA is the preferred immunoassay method as it is more accurate and less time-consuming than other available methods (such as radioimmunoassay and Western blot analysis). ELISA uses a combination of antibodies to detect secreted insulin. The primary antibody should be a monoclonal antibody to achieve the best sensitivity and should have an insulin-specific epitope that binds to insulin. The secondary antibody may be a monoclonal or polyclonal antibody that attaches to the primary antibody. The enzyme is usually linked to a second antibody. Enzymes convert substrates into fluorescent or bioluminescent products, which can then be detected by appropriate instruments. An ELISA-based insulin secretion assay begins with seeding a number of pancreatic β-cells with a culture medium in the 96-welled plate. Cells grow under the required conditions (e.g. 5% CO_2_ at 37°C). After the cells have adhered to the plate, they should be washed with Krebs-Ringer bicarbonate (KRB) buffer containing a lower glucose concentration than conventional media, as this step requires cell starvation. KRB buffer containing NaHCO_3_, CaCl_2_, KCl, NaCl, MgSO_4_, KH_2_, PO_4_, 4-(2-hydroxyethyl)-1-piperazineethanesulfonic acid, d-glucose, and bovine serum albumin, at a pH of 7.4, is used as a starvation buffer [24]. The time of starvation should be optimized because it is one of the critical steps in this assay. Longer starvation times can kill cells, while short times will lead to inconsistent results. Typically, the starvation period used for this assay is 60-90 minutes. After starvation is complete, the compound or extract should be treated with a KRB buffer containing a higher glucose concentration than conventional media. Treated cells should be left in a CO_2_ incubator to secrete insulin. Exceeding the incubation time may reduce insulin levels to basal levels. After this process, the medium containing secreted insulin is collected to quantify the exact amount of insulin. Therefore, the ELISA method is used according to the manufacturer's instructions. There is a variety of ELISA kits available, including human insulin ELISA, rat/mouse insulin ELISA, rapid insulin ELISA, and others. The choice of the most suitable kit should be based on the source of insulin, protocol, and sensitivity of the kit [24, 25].

The luciferase-based assay is based on the enzyme luciferase or luminescence. This assay is discussed in section 3.2 on the reporter gene assay. It is a universal assay because it can be used for both low and high throughput. For screening or identification purposes, it can also be used with crude extracts and compounds. In luciferase-based assays, the gene of luciferase, either Guassia or Firefly, is incorporated in the C-peptide of pro-insulin. After successful insertion of these genes, the expression of insulin and luciferase genes and the secretion of insulin and luciferase should be checked [26]. If both expression and secretion are in close correlation and higher than non-treated cells, then it can be concluded that the test compounds are responsible for the upregulation of the insulin gene [26].

### 3.4 Calcium measurement assay

Calcium plays an important role in bone mineralization and a wide range of biological functions. Ca^2+^ mobilization is an indirect method to measure the effect of compounds/drugs on g-protein-coupled receptors (GPCRs) [33]. Ca^2+^ mobilization leads to the activation of GPCR, which affects phospholipase-C (PLC) that releases inositol phosphate 3 (IP3). Intracellular Ca^2+^ mobilization occurs in the endoplasmic reticulum and eventually affects various cellular processes. cAMP is the main key element that regulates insulin secretion and also provides a mechanism for intracellular Ca^2+^ storage in islet cells [33]. Some reports indicate that glucose-dependent characteristics are observed in insulin secretion regulated by cAMP. For example, during bioassay and ELISA tests of extracts of *Leonurus sibiricus L*, caffeine, and betulinic acid compounds, it has been shown that direct or indirect release of intracellular Ca^2+^ may lead to insulin secretion [34].

Ca^2+^-chelating agent can directly measure Ca^2+^ content [34]. Ca^2+^-chelating agent, i.e. the fluorescent dye, combined with Ca^2+^, increases the obvious fluorescence intensity after the dye is combined with Ca^2+^ and measured by a fluorometer. Commonly used classic dyes include Fura-2, Indo-1, Fluo-3, and Fluo-4 [35]. Fura-2 dye (fluorescent calcium indicator) is used to monitor intracellular calcium levels in insulinoma 1 (INS-1) cells. Cells seeded on poly-L-ornithine-coated coverslips should be incubated in culture dishes for 72 hours. After incubation, cells should be loaded with 1 mM Fura-2 in modified Krebs-Ringer bicarbonate HEPES buffer (KRBH). An extracellular solution containing no Ca^2+^ should be prepared by excluding CaCl_2_ and adding 2 mM EGTA. Fura-2 dye should be removed followed by washing and incubation of the cells with KRBH buffer for 30 minutes at room temperature. Samples containing coverslip should be installed in the perfusion chamber on the fluorescence microscope stage. The fluorescence intensity is detected at an excitation wavelength of 340-380 nm and an emission wavelength of 510 nm. According to the principle, the fluorescence intensity is proportional to the Ca^2+^ level. This is a straightforward and time-saving method for detecting intracellular Ca^2+^ levels. The only drawback of this method is that the background signal from the cell-free area is recorded as the Fura-2 dye is a double excitation dye. The background signal can be invalidated by narrowing down the excitation beam [35].

### 3.5 ATP measurements assay

The measurement of intracellular ATP is a quantitative process called ATP measurement analysis. It is used to measure the level of ATP in the cell. In this assay, luciferase is commonly used to detect or release ATP. This enzyme catalyzes ATP-dependent D-luciferin into oxidized luciferin, which emits light at 560 nm. Therefore, the light emitted is proportional to the number of luciferase molecules [38]. The stock solution should be prepared by dissolving ATP in deuterium-depleted water and then diluting it in 1 liter of deuterium-depleted water. Standard ATP should be prepared by diluting the ATP stock solution in polystyrene sulfonate.

To prepare the fluorescein mixture, fluorescein is dissolved in deuterium-depleted water. Standard ATP should be prepared by diluting the ATP stock solution in polystyrene sulfonate. To prepare the luciferin mixture, luciferin is dissolved in deuterium-depleted water. Therefore, 5 ml of the mixed solution is poured into a vial containing a solid mixture of luciferin. To calculate the amount of ATP released, the luciferin mixture should be placed in a 500 μl syringe and the ATP standard solution should be placed in another syringe. Both solutions should be pumped through a microcentrifuge tube of 30 cm per section. The streams containing the luciferin mixture and the ATP standard are combined at the hybrid T-junction. Photomultiplier tubes (PMT) are used to record the results of chemiluminescence which is generated by reaction ATP [39].

### 3.6 cAMP assay

**Figure 3.**
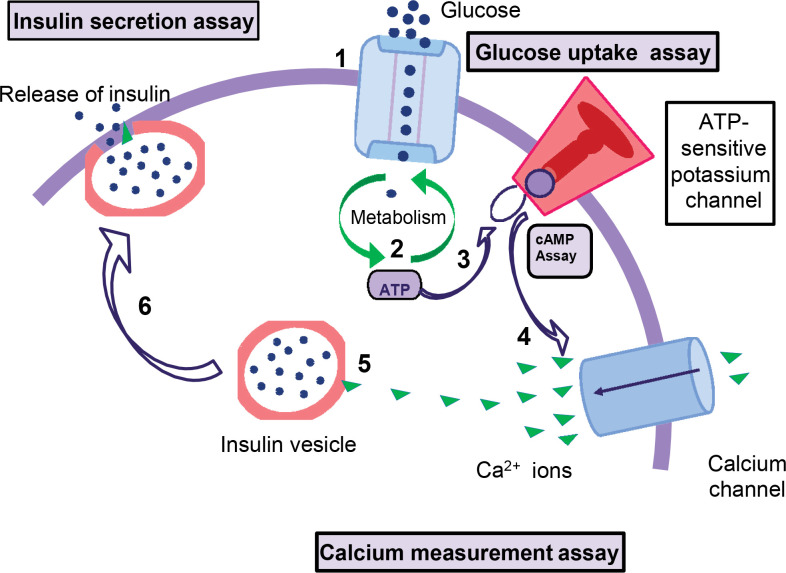
**Mechanism of the cAMP-dependent pathway of insulin secretion**. 1. Increased blood glucose levels can lead to glucose uptake (glucose uptake assay). 2. Intracellular glucose metabolism occurs, the ATP/ADP ratio increases, and cAMP levels also increase (cAMP assay and ATP measurement assay). 3. and 4. Blocking ATP-sensitive potassium channels causes depolarization of the cell membrane and releases calcium ions through calcium channels (calcium measurement assay). 5. Calcium combined with insulin vesicles results in insulin secretion (determination of insulin secretion).

In diabetes, cAMP plays a very critical role in insulin secretion. The conversion of ADP to cAMP releases ATP molecules that bind to the ATP-sensitive potassium channel on pancreatic β-cells and cause the channel to close [40]. The membrane depolarizes and then opens to allow the intracellular flow of calcium channels [41]. Calcium ions enter β-cells, causing insulin to be released from the vesicles. Therefore, the level of cAMP is directly proportional to the level of insulin. The mechanism of the cAMP-dependent pathway of insulin secretion is explained in Figure 3.

The assay starts with incubation of INS-1 cells in Krebs buffer with a range of glucose concentrations [42]. It also contains vanillic acid in various concentrations [43]. In the presence of glucose, the cells should be stimulated with different concentrations of vanillic acid under gentle shaking for 30 minutes. In several experiments, before adding 50 μM vanillic acid, INS-1 cells should be preincubated in 0.25 mM isobutyl methylxanthine and 10 μM SQ (inhibitor of adenylate cyclase) [44, 45]. Intracellular cAMP levels are determined by enzyme immunoassay kits [43, 46, 47]. Advantages include continuous real-time monitoring and high-throughput detection [48].

## 4 Target-based assay

Target-based measurement of insulin secretion is performed when the defined target and specific target of the molecule are known. Compared with the phenotyping method, it has the advantages of being accurate, precise, specific, and fast. It is very useful to understand the mechanism of action of compounds.

### 4.1 PPARγ and GLUT-4 assay

The transcription factor PPARγ is one of the main targets of antidiabetic drugs. Thiazolidinedione is a drug used for the treatment of diabetes and other insulin-resistant diseases and targets PPARγ. PPARγ and GLUT-4 are present in adipose tissue and muscle cells [49]. They are interconnected with each other. When PPARγ binds to its receptor, it increases the expression of the GLUT-4 gene in these tissues. This increase in expression is responsible for glucose uptake. The expression of both genes indicates the effect of any compound on these targets.

To determine the effect of any compound or crude extract on PPARγ and GLUT4 expression, cells should be treated with this compound for 24 hours. RNA isolation is the next step, which can be performed with Triazole Reagent or any other method [50]. A reverse transcription step should be performed to convert the mRNA to cDNA. Then qPCR should be performed to amplify the DNA. Before amplification, the transcriptase should be inactivated. Primers should be specific for the genes of PPARγ and GLUT-4. The threshold cycle value (i.e. Ct and **Δ**Ct) should be measured. Changes in the expression level (ΔΔCt) relative to other genes should be considered. Increased GLUT4 gene expression will lead to increased glucose uptake [51, 52].

### 4.2 Alpha-amylase assay

Alpha-amylase is a major enzyme that originates in saliva and pancreatic fluid and can break down sizeable insoluble starch molecules into absorbable molecules. Inhibition of α-amylase in the pancreas is the main therapeutic goal of ensuring that oligosaccharides are digested into absorbable monosaccharides in the intestine's brush border area which is able to reduce postprandial hyperglycemia subsequently [53].

The 3,5-dinitrosalicylic acid (DNSA) method is a well-known method for α-amylase inhibition assays. This technique works by first dissolving the sample in dimethyl sulfoxide and then in buffers of different concentrations (Na_2_, HPO_4_, NaCl at pH 6.9). Also, a volume of α-amylase solution should be mixed with a volume of the sample and incubated at 30°C for 10 minutes. Then, each test tube filled with the starch solution should be incubated for 3 minutes. Next, DNSA reagent (potassium sodium tartrate tetrahydrate in 2M NaOH and 96 mM 3,5-DNSA solution) should be added and boiled for 10 minutes. The mixture should be cooled down and diluted with distilled water [54, 55]. UV-visible spectrophotometer should be used for the measurement of absorbance (at 540 nm). The blank solution should be prepared by replacing the sample with buffer; the solution is considered to have 100% enzyme activity [56]. A blank reaction should also be prepared using samples of each concentration of the enzyme-free solution. Acarbose is an inhibitor of alpha-amylase used as a positive control [54]. Alpha-amylase inhibition percentage should be assessed by the formula: {(absorbance (control) - absorbance (sample))/ (absorbance (control))} x 100 [57].

### 4.3 Alpha-glucosidase assay

Alpha-glucosidase is located in the brush border of the small intestine area. It is able to assist the digestion of dietary carbohydrates and inhibits postprandial hyperglycemia [58]. The alpha-glucosidase assay consists of a reaction mixture, including a 20 mM p-nitrophenyl-α-glucopyranoside (PNPG) solution containing p-nitrophenol (PNP), glucose, and 0.2 M sodium carbonate solution. The different concentrations of samples/extracts should be dissolved in 100 mM potassium phosphate buffer at pH 6.3. The control tube contains only DMSO, enzyme, and substrate, while in the positive control tube, acarbose replaces the sample/extract. Without enzymes, a mixture of sample extract and acarbose is used as a blank. The entire reaction mixture is to be incubated at 30°C for 5-6 minutes. After completion of incubation, the reaction is terminated by adding 2.0 ml of Na_2_CO_3_ solution. Using a spectrophotometer, the absorbance rate measured at 400 nm is proportional to the activity of the enzyme [59, 60].

### 4.4 PTP1B assay

The phosphoprotein tyrosine 1B (PTP1B) assay is a colorimetric-based assay that plays an essential role in insulin cascade signaling [58]. Therefore, in general, PTP1B analysis involves the dephosphorylation of charged insulin receptors. It leads to inactive glucose uptake [61]. Inhibitors of PTP1B can minimize insulin resistance. In general, the IC_50_ values of natural 1.5 μM to 30 μM and semi-synthetic inhibitors are 1 mM to 12 μM [62]. The well-known substrate p-nitrophenyl phosphate (pNPP) is generally used to evaluate the activity of the PTP1B enzyme. This assay should be performed by adding 10 μl of the test compound solution and 20 μl of an enzyme (1 μg/ml) and mixing 40 μl of 4 μM pNPP with 130 μl of the given buffer (Tris-HCl, β-mercaptoethanol, EDTA, and DTT) at 37°C [63, 64].

During enzymatic reaction, the amount of p-nitrophenolate produced by enzymatic dephosphorylation of pNPP should be evaluated by calculating the absorbance at 405 nm using a microplate reader spectrophotometer. Ursolic acid should be used as a positive control for inhibition [65]. Sensitivity is an advantage of this method [58].

## 5 Conclusions

Significant advances have been made in technologies to empower assay measurements for T2D with improved automatization, throughput, and data management. As far as early drug discovery is concerned, *in-vitro* assays can help to determine whether the compound is acting on the target molecule. They can provide insight into the effectiveness of the compound and whether it has potentially harmful toxic effects. The screening of these target molecules/extracts using *in-vitro* assays is accomplished by two approaches that differ in their initial focus, namely phenotypic assay and target-based assay.

**Figure 4.**
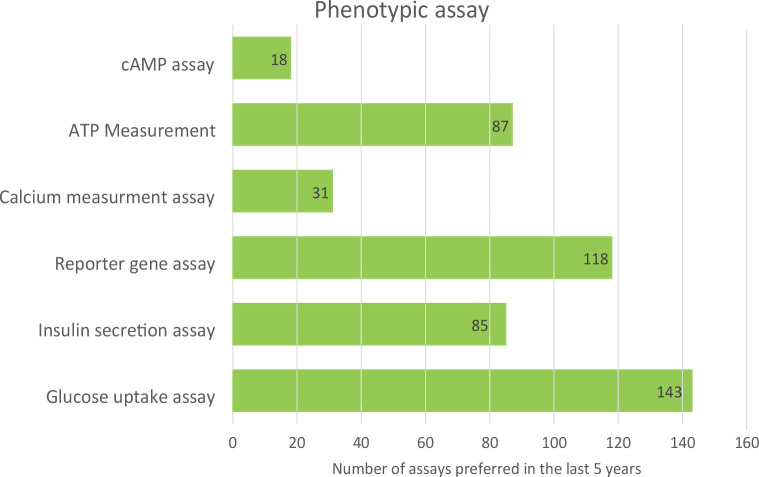
Popularity of different phenotypic-based assays.

**Figure 5.**
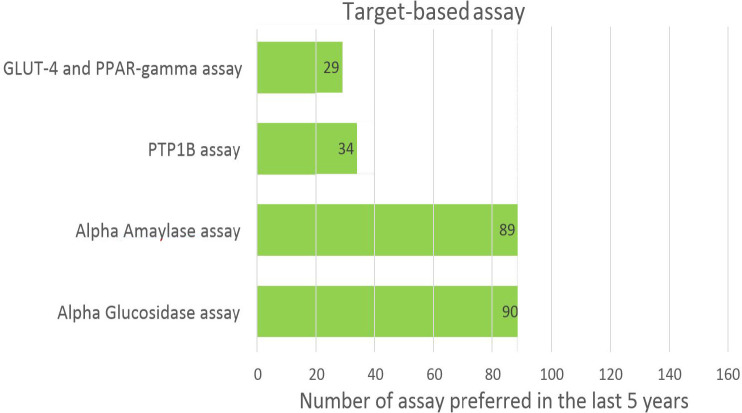
Popularity of different target-based assays.

Phenotypic assays are useful at the early stage of drug discovery when the mechanism of molecules is unknown, and they are used for extract activity validation. In contrast, target-based assays have focal points; they are simpler to execute, faster, easier, and more accurate and specific. In this review, we have comprehensively classified *in-vitro* assays into two categories. Figure 4 and 5 show the usage frequency of phenotypic and target-based assays. As can be seen from Figure 4, glucose uptake assays have attracted the most interest among phenotypic assays; this is due to their high selectivity and optimal signal-to-noise ratio.

Among target-based assays, α-amylase and α-glucosidase assays are most frequently used since they are easy to use for screening of compounds inhibiting the enzyme α-amylase and α-glucosidase. Table 1 shows the advantages, limitations, and quality parameters of all assays. These data may be useful at various stages of drug discovery for T2D, ranging from hit identification to lead optimization.

**Table 1.** Advantages, disadvantages, applications, and quality parameters of *in-vitro* assays

Assay	Subpart	Advantages	Disadvantages	Applications	Quality parameter
Glucose uptake assay	Radiolabeled	More selective, better signal-to-noise ratio	Costly, difficult to handle radiolabeled material, requires specific condition, hazardous	For crude extracts and compounds	Positive control: insulin
	Non-Radiolabeled	Easy to handle, high throughput possible	Sensitivity based on the detection techniques and instruments	High throughput screening, for crude extracts and chemical compounds	
Insulin secretion assay	ELISA based assay	Sensitive, Easy to use	Expensive temporar y readouts	For crude extracts, synthesized compounds, and isolated compounds also	Positive control: glibenclamide (hypoglycemic drug) Negative control: non-treated cells
	Luciferase based assay	High-throughput, no need for exogenous illumination, sensitive, uantitative	The substrate should be provided	For crude extracts, synthesized compounds, and isolated compounds as well	Housekeeping gene
Reporter gene assay		High throughput, quantitative	Sensitivity depends on the type of reporter gene	For compounds that affect gene regulation	Endogenous control- housekeeping gene: beta actin
Calcium measure- ment assay		Simple and less time consuming	High background fluorescence	-	Control: solvent-treated cells positive control: ionomycin or TCDD (2,3,7,8-Tetrachloro- dibenzo-p-dioxin)
ATP mea- surement assay		Less time consum- ing, user-friendly, reliable and accurate	Chances of false positive, Sensitive	-	Control: housekeeping gene
cAMP assay		Robust, good for agonist and antagonist discrimination	Radioactive Chances of false results	Cell growth rates can be determined	Wortmarin in DMSO
PPAR**γ**and GLUT-4 assay	Type of reporter gene assay	Easy to determine the compounds with this target	Not used for any other molecules which do not have the PPARɤ and GLUT-4 target	For compounds that affect PPARɤgene expression	Positive control: piogl- itazone Negative control: non-treated cells
Alpha-amy-lase assay		Easy enzymatic assay for compounds that inhibit alpha-amylase	Limit of detection is very low	Used for compounds with the inhibition mechanism of alpha-amylase	Untreated cells
Alpha-glu-cosidase assay		Easy enzymatic assay for compounds that inhibit alpha-glucosidase	Not for other compounds	Use for the compounds with the inhibition mechanism of alpha-glucosidase	Untreated cells
PTP-1B		Convenient, not radioactive	-	-	Acarbose dissolved in DMSO

## Abbreviations

2DG 2-deoxy-D-glucose

DG6P 2-deoxy glucose 6-phosphate

2NBDG 2-[N-(7-nitrobemz-2-oxa-1,3- diazol-4-yl) amino]-2-deoxyglucose

3MG 3-methylglucose

DNSA 3,5-dinitrosalicylic acid

6NBDG 6-[N-(7-nitrobemz-2-oxa-1,3-diazol-4-yl) amino]-2-deoxyglucose

ABCC8 ATP-binding cassette transporter sub-family Cmember 8

AMPK AMP-activated protein kinase

DAG diacylglycerol

G6PDH glucose-6-phosphate dehydrogenase

GFP green fluorescent protein

GLUT4 glucose transporter 4

GPCR G-protein-coupled receptor

IDF International Diabetes Federation

INS-1 insulinoma 1

IP3 inositol phosphate 3

KRB Krebs-Ringer bicarbonate

KRBH Krebs-Ringer bicarbonate HEPES

KCNJ11 potassium inwardly rectifying channel subfamily J 11

NADP+ nicotinamide adenine dinucleotide phosphate

PLC phospholipase C

PMT photomultiplier tubes

pNP p-nitrophenol

pNPG p-nitrobenzene-α-glucopyranoside

pNPP p-nitrophenyl phosphate

PPARɤ peroxisome proliferator-activated receptor γ

PPARα peroxisome proliferator-activated receptor α

PTP1B phosphoprotein tyrosine 1B

RIA radioimmunoassay

T1D type 1 diabetes mellitus

T2D type 2 diabetes mellitus

YFP yellow fluorescent protein
